# The association of symptoms of overactive bladder with pelvic organ prolapse and its improvement after pelvic reconstructive surgery

**DOI:** 10.12669/pjms.37.3.3312

**Published:** 2021

**Authors:** Saida Abrar, Raheela Mohsin Rizvi, Nida Zahid

**Affiliations:** 1 Dr. Saida Abrar, Department of Gynecology/Obstetrics, Clinical Fellow Urogynecology and Pelvic Reconstructive Surgery, Aga Khan University Hospital, Karachi, Pakistan; 2 Dr. Raheela Mohsin Rizvi, Section of Urogynecology, Clinical Fellowship Urogynecology Sydney, Australia, Aga Khan University Hospital, Karachi, Pakistan; 3 Dr. Nida Zahid, Department of Surgery, Senior Instructor Research, Acting Head of Data Monitoring and Evaluation Division Office of Academia and Research, Aga Khan University Hospital, Karachi, Pakistan

**Keywords:** Overactive bladder (OAB), Pelvic organ prolapse (POP), Pelvic organ prolapse surgery

## Abstract

**Objectives:**

This study asseses the association of overactive bladder symptoms and pelvic organ prolapse severity and evaluates the effect of pelvic reconstructive surgery on overactive bladder (OAB) symptoms in women with pelvic organ prolapse (POP). It also looks into any pre and post-operative factors responsible for persistent postoperative OAB symptoms.

**Methods:**

This was a retrospective cross-sectional study conducted at the Aga Khan University Hospital, Karachi between 1st January 2014 and 31st December 2018. In this study women presenting with POP and concommitent OAB who underwent surgery for site specific defects, measured using Pelvic Organ Prolapse Quantification (POP-Q) staging system. OAB was defined as presence of urinary frequency, urinary urgency incontinence (UUI) and an affirmative response to item #15 and/or item #16 of the Pelvic Floor Distress Inventory (PFDI), which was used both pre and postoperatively. Primary outcome of the study was to find complete resolution or improvement of urinary frequency and UUI on the PFDI, 24 months after surgery. The secondary outcome was to see persistent OAB postoperatively and the factors associated with it.

**Results:**

Overactive bladder (OAB) symptoms improved significantly regardless of the severity of prolapse at 24 months postoperative period. Body mass index (BMI) and postoperative constipation were the only statistically significant variables associated with persistent OAB symptoms postoperatively.

**Conclusions:**

Surgical correction of POP results in significant improvement in symptoms of OAB, in all stages of POP and co-existing OAB. However women with high BMI and post-operative constipation may be prone to persistent frequency and/or UUI.

## INTRODUCTION

Pelvic Organ Prolapse (POP) is a common disorder worldwide and is defined as “Descent of one or more of the anterior, posterior vaginal wall, the uterus (cervix) or the apex of the vagina (vaginal vault or cuff scar after hysterectomy)”.^1^ The reported worldwide prevalence of POP is 9%,^2^ and about 20% in low-income countries.^3^ According to the International Continence Society (ICS), OAB is defined as “urgency with or without urgency urinary incontinence, usually with frequency and nocturia in the absence of urinary tract infection (UTI) or other obvious pathology”.^1^ Whether POP should be considered as ‘obvious pathology’ is still a debatable issue. Both of these conditions usually co-exist, thus affecting the diagnosis of these diseases and their possible treatment options. The Integral Theory states that OAB and detrusor overactivity is caused by a normal but prematurely activated micturition reflex due to poor support of stretch receptors of the bladder base caused by a lax vagina.^4^ Communities as well as hospital studies have shown high prevalence of OAB symptoms in patients with POP with a significant effect on the psychological, economic profile and overall quality of life of a woman.^5^ Literature demonstrates heterogeneous results regarding improvement in symptoms of OAB after surgery for POP.^6^ Regional data on this topic is lacking, however a study in Japan showed improvement in lower urinary tract symptoms (LUTS) in most of the women with POP after pelvic reconstructive surgery using the transvaginal mesh and mid-urethral sling (MUS) procedure.^7^ There are no studies specifically addressing the association of OAB of OAB and POP severity in Pakistan, although a regional study from Turkey showed that grand-multiparity and vaginal delivery increases the risk of surgery for POP and/or SUI.^8^

There is a gap in literature regarding relationship of symptoms of OAB and preoperative grades of POP and also about the factors associated with persistent OAB symptoms after surgery for POP.^9^ The aim of this study was to find the improvement in symptoms of OAB after pelvic reconstructive surgery using PFDI-20. We also evaluated any pre and postoperative factors leading to persistence of urinary frequency and UUI after pelvic reconstructive surgery.

## METHODS

We conducted a retrospective cross sectional study at the Aga Khan University Hospital, Karachi between 1st January 2014 and 31st December 2018. One hundred and thirty-five women with POP and coexisting OAB for at least six months duration were included, who had undergone vaginal hysterectomy, anterior colporrhaphy and posterior colpoperineorrhaphy using native tissue and one additional procedure for vault suspension i.e Mcalls culdoplasty.

Variables like demographic data, clinical characteristics and record of physical examination were collected on structured proformas. All women were evaluated by simplified POP-Q staging performed as described in the POPQ system by the ICS,^10^ cough test, urine analysis to rule-out urinary tract infection, ultrasound for post-void residual and uroflowmetry. Preoperatively POP was grouped as stage 1 to 2 and stage 3 to 4 using the POP-Q system, where POP stage 1–2 is less severe and stage 3–4 is more severe anatomical groups.^11^ For evaluation of symptoms of OAB and POP, we used PFDI-20 both before and on each follow up visits after surgery. The follow up schedule at our hospital includes first visit one week postoperatively, then after 3, 6, 12, 24 months of surgery and thereafter in case of any symptoms only. We excluded twenty patients from the study. These patients either had concommitant surgery for stress urinary incontinence (SUI), did not complete the PFDI-20 questionnaires or a complete physical examination was not available. All the patients were operated by same surgeon and had postoperative follow up for at least two years.

OAB symptoms were defined as the presence of symptoms of urinary frequency and UUI and an affirmative response to item no15 and/or item no16 on PFDI-20.^12^ The PFDI-20 uses a five-point Likert scale, with the higher numbers representing more severe symptoms, where 1 represent- Not at all, 2: Somewhat, 3: Moderately and 4: Quite a bit, while 0 representing no symptoms of OAB.

We evaluated the effect of baseline severity of POP on improvement in symptoms of OAB, 24 months after pelvic reconstructive surgery. Any pre and postoperative factors leading to persistence of symptoms of urinary frequency and/or UUI were also determined by analyzing demographic characteristics and preoperative POP severity. Postoperative improvement was defined as either complete resolution of symptoms of urinary frequency and UUI (symptoms present preoperatively, absent postoperatively), or improvement as shown by a decrease by at least one number in the severity of symptom.

For descriptive data analysis, qualitative data like co-morbid, urinary frequency, UUI was presented as frequencies and proportions while quantitative variables such as age, parity, BMI were presented as means and standard deviations. For inferential statistics, continuous scale data was analyzed using Students t-tests, Man Whitney U-test, Wilcoxan-sign rank test while Chi-squared test or Fisher’s exact test was used to compare categorical data. The analysis was carried out using SPSS package version 21 (IBM Corp.; Armonk, NY, USA) and p-value ≤ 0.05 was taken as statistically significant.

### Ethical approval:

The study was conducted after approval from hospital ethical review committee (ERC-2019-1573-4180, dated July 7, 2019).

## RESULTS

We included one hundred and thirty-five patients in the study, out of which seventy-two had anatomically less severe and sixty-three had anatomically more severe degree of pelvic organ prolapse. The baseline characteristics of the patients are listed in Table-I. Women with stage 3-4 POP had a significantly higher mean age (62.108±10.54) as compared to the less severe POP group (59.08± 9.82) (p=0.001). However there was no significant difference in the BMI, duration of overactive bladder symptoms, use of anticholinergics, parity and co-morbids. Aa, Ba, Ap, Bp, C and D points (by POP-Q) were significantly higher in the more severe anatomical prolapse group than the less severe anatomical prolapse group (P<0.001) (Table-I). We also observed significant improvement in frequency and UUI after pelvic reconstructive surgery in both groups (P<0.001) (Table-II).

**Table I:** Baseline Characteristics of patients with Pelvic organ prolapse.

	Pelvic organ prolapse (Stage 1 and 2) (n=72)	Pelvic organ prolapse (Stage 3 and 4) (n=63)	p-value
Age (years) (Mean±SD)	55.67 (9.82)	62.108 (10.54)	<0.001*
Parity (median (IQR))	4 (3-5)	4(3-5)	0.813
BMI (kg/m2) (median(IQR))	26.5 (23.6-32)	25. 4(23.6-31.6)	0.637
Duration of symptoms (months) (median (IQR))	10 (8-12)	12 (8-12)	0.338
***Use of anticholinergics***			
No	49 (80.3%)	53 (71.6%)	0.241
Yes	12 (19.7%)	18 (28.4%)	
***OAB symptoms more than 12 months n(%)***			
No	31 (50.8%)	41 (55.4%)	
Yes	30 (49.2%)	33 (44.6%)	0.595
***HTN***			0.552
No	27 (44.3%)	29 (39.2%)	
Yes	34 (55.7%)	45 (60.8%)	
***DM***			
No	41 (67.2%)	43 (58.1%)	
Yes	20(32.8%)	31 (41.9%)	0.278
***Hypothyroid***			
No	58 (95.1%)	67 (90.5%)	0.511
Yes	3 (4.9%)	7 (9.5%)	
Aa point (cm) (median (IQR))	1	6 (3-7)	<0.001*
Ba- point(cm) (median (IQR))	1	6 (4-7)	<0.001*
Ap point (cm) (median (IQR))	0 (0-1)	4 (2-7)	<0.01*
Bp point (cm) (median (IQR))	0 (0-1)	4 (2-7)	<0.001*
C point (cm) (median (IQR))	1 (0-1)	5 (2-7)	<0.001*
D point (cm) (median (IQR))	8	6 (4-8)	<0.001*

Data are mean±standard deviation, median ( interquartile range (IQR)) or number (%), ***BMI:*** Body Mass Index, ***HTN:*** Hypertension, ***DM:*** Diabetes mellitus,

* significant at p-value <0.05 by using Independent t-test / Mann whitney U test

**Table II:** Comparison of changes in the overactive bladder symptoms in both groups of POP before and after surgery.

	Preoperative		Postoperative		P-value
Frequency	2(2-3)	1±1-2	1(1-2)	1±0.00	0.001*
Urge incontinence	2(2-3)	1±1-2	1(1-2)		0.001*

Data are median ( interquartile range (IQR)); Data was defined by Pelvic Floor Distress Inventory item no. 15 (frequency) and item no. 16 (urge incontinence

* significant at p value < 0.05 by using wilcoxin sign rank test

Table-III shows that proportion of improved patients is similar in both high and low POP groups. Table-IV indicates pre and postoperative factors associated with persistence and worsening of urinary frequency and UUI after surgical repair of POP. We observed that the frequency symptoms persisted in females with higher 30.7(24-35) versus those who had lower BMI 25 (24-28) (p=0.003), similarly UUI persisted in females with higher 31.2 (25-37) versus those with lower BMI 25.4 (24-29) (p- value=0.001). We also observed that UUI persisted in significantly higher proportion of patients (70.3%) with postoperative constipation versus those who had no postoperative constipation (29.7%) (p=0.004). However there was no significant association between age, parity and other variables with persistent OAB symptoms.

**Table III:** Overactive bladder symptoms before and after surgery.

	POP stage 1 and 2 (n=72)	POP stage 3 and 4 (n=63)	P-value
***Frequency***			
Improvement	28 (45.9%)	35 (47.3%)	
Resolution	7 (11.5%)	8 (10.8%)	0.757
Persistence	14 (23.0%)	21 (28.4%)	
Worsen	12 (19.7%)	10 (13.5%)	
***Urge incontinence***			
Improvement	26 (42.6%)	37 (50.0%)	
Resolution	14 (23.0%)	16 (21.6%)	
Persistence	18 (29.5%)	16 (21.6%)	0.707
Worsen	3 (4.9%)	5 (6.8%)	

Data are number (%).POP-pelvic organ prolapse. Urinary frequency defined by Pelvic Floor Distress Inventory item no. 15; urge incontinence defined by Pelvic Floor Distress Inventory item no. 16.

**Table IV:** Pre and postoperative factors related to persistence and worsening of overactive bladder symptoms after POP repair.

	Frequency (n=78)			Urge incontinence (n=57)		

	Improvement^a^ n= 78 (57.8%)	Persistence^b^ n=57 (42.2%)	p-value	Improvement n=98 (72.6%)	Persistence n=37 (27.4 %)	p-value
Age (yr) Mean ±SD	60 ± 10	57.9 ± 10	0.233	58.87 ± 11	60.05 ± 9.8	0.570
Parity Median (IQR)	4 (3-6)	4 (3-5)	0.28	4 (3-5)	5 (3-6)	0.139
Ba point (cm) Median (IQR)	2 (1-6)	2 (1-6)	0.758	2 (1-6)	1 (1-5)	0.151
Bp point (cm) Median (IQR)	1 (0.0-4)	1 (0.0-4)	0.886	1 (0.0-4)	1 (0.0-3.5)	0.122
C point Median (IQR)	1 (0.0-4)	1 (0.0.4.5)	0.829	1.50 (1-6)	1 (0.0-4)	0.181
BMI (kg/m2) Median (IQR)	25 (24-28)	30.7(24-35)	0.003*	25.4 (24-29)	31.2 (25-37)	0.001*
***POP stage n(%)***						
Stage 1 and 2	35 (44.9%)	26 (45.6%)	0.932	40 (40.8%)	21 (56.8%)	0.097
Stage 3 and 4	43 (55.1%)	31 (54.4%)		58 (59.2%)	16 (43.2%)	
***Post-operative constipation n(%)***						
Yes	41 (52.6%)	26 (45.6%)	0.425	42 (42.9%)	26 (70.3%)	0.004**
No	37 (47.4%)	31 (54.4%)		56 (57.1%)	11 (29.7%)	

Data are mean ± standard deviation, median (IQR) or number (%).

a Women with resolution of symptoms were included in the improvement group

b Women with worsening of symptoms were included in the persistence group. POP- pelvic organ prolapse; BMI-body mass index,

* significant at p value < 0.05 by using Mann whitney U test,

** significant at p value < 0.05 by using chi-square test

## DISCUSSION

This study shows a significant improvement in symptoms of urinary frequency and UUI after pelvic reconstructive surgery for symptomatic POP in women with co-existing OAB. However this improvement was not statistically different between the anatomically less severe and more severe stages of POP. Preoperative BMI and postoperative constipation was associated with persistant OAB symptoms. Many studies have demonstrated a clear association between symptoms of OAB and POP.^13^

Treatment of POP, whether conservative or surgical is associated with an improvement in OAB symptoms. The exact mechanism is not established but one of the proposed mechanisms is bladder outlet obstruction.^14^,^15^

In a study by Fletcher et al, it was found that symptoms of OAB assessed by using UDI-6, were improved after surgery equally in both groups of stage 1-2 & stage 3-4 POP.^16^ However only patients with anterior wall defects corrected by anterior colporrhaphy were included in this study. Similarly a study by Digesu et al. demonstrated significant improvement in OAB symptoms and quality of life, one year after anterior vaginal wall repair assesed by King’s Health Questionaire and Prolapse Quality of Life questionnaire.^17^ In another study by Mi Sun Kim et al, similar improvement in OAB symptoms was observed in all stages after POP surgery but they did not find any statistically significant preoperative factors leading to persistence of symptoms of OAB postoperatively.^10^

In contrast to our study, Miranne et al. observed greater improvement in OAB symptoms after reconstructive surgery for lesser degree of POP. His study revealed that improvement in frequency and UUI was more in patients with stage 1-2 POP as compared to stage 3-4 POP (Frequency 89% versus 85% and UUI 90% versus 85%).^18^ They included patients with uterine prolapse or those having anterior vaginal wall prolapse and only preoperative POP stage was used for predicting the variables associated with persistent postoperative symptoms of OAB, without considering any pre or postoperative demographic factors.

OAB comprises of spectrum of multiple symptoms and in our study we considered only symptoms of frequency and UUI but in another prospective study by de Boer et al, full spectrum of OAB was taken including Urinary frequency, urgency, nocturia and UUI. The study showed more improvement in preoperative frequency and urgency than UUI or nocturia after POP repair.^19^ In contrast to our study the improvement in urinary frequency and urgency was greater with advanced stage of POP. The author attributed this to denervation of the the detrusor muscles leading to permanent changes in the bladder due to chronic POP. However this study did not consider factors that may lead to persistence of these symptoms.

The evaluation of POP does not include urodynamic studies (UDS) however it is recommended that UDS should be performed in the presence of LUTS and asymptomatic POP. We considered clinical diagnosis of OAB and a diagnosis of neurogenic bladder was excluded after UDS. Romanzi et al.^20^ revealed that as compared to grade 1-2, grade 3 to 4 cystocele was associated with a much higher rate of bladder outflow obstruction (BOO) (58% versus 4%) and detrusor overactivity (52% versus 20%) on urodynamics. Surgical repair of POP resulted in resolution of BOO in ninetyfour percent of women with grade 3-4 cystocele. POP surgery also included transvaginal mesh and concomitant sling procedures.

The use of antimuscarinic drugs and constipation is well established, however majority of patients in our study, both in the low and high grades did not use antimuscarinic drugs. This study finds BMI and postoperative constipation as the only factors related to persistent OAB symptom after pelvic reconstructive surgery in contrast to a retrospective study of 203 patients, which found old age as a risk factor for persistent OAB symptoms.^21^,^22^

Points of strength of our study is that all patients had similar surgical procedure of vaginal hysterectomy with anterior colporrhaphy and posterior colpoperineorrhaphy using native tissue, by the same surgeon. There were sufficient number of patients in both the less and advanced stages of POP making it possible to obtain more robust findings. Postoperative data was taken from patients medical records, thus both subjective and objective data of pelvic anatomy postoperatively was available. Since we followed patients for a mean period of thirty four months postoperatively, hence making us able to evaluate persistence, improvements or deterioration in symptoms of OAB over a long time.

### Limitations of the study:

This study has certain limitations such as its retrospective design. However Health Information Management Services (HIMS) of AKUH is structured computerized database that maintains patient data in a systematic way. This study was unable to address changes in OAB symptoms after site specifics defects correction for POP. We were also unable to measure urgency as it is not included in PFDI-20, thus missing the possibility of including women with dry OAB.

## CONCLUSION

Pelvic reconstructive surgery for POP also results in significant improvement in the associated OAB symptoms in all stages of POP. Preoperative factors like BMI and post-operative constipation are risk factors for persistent OAB after surgery for POP in patients with coexisting OAB and POP. This information may be helpful in counseling of women with POP and OAB symptoms, who are planned for pelvic reconstructive surgery. To increase the strength of evidence, future prospective studies are required to find association of OAB with any specific compartment and to confirm our findings of factors responsible for persistent OAB.

### Authors` Contribution:

**SA** conceived, designed, data collection and manuscript writing and is responsible for integrity of the study.

**RR** did editing and final approval of manuscript, accuracy and integrity of the work.

**NZ** did statistical analysis.
